# Workforce Characteristics of Med-Peds Hospitalists

**DOI:** 10.7759/cureus.24799

**Published:** 2022-05-07

**Authors:** Roma Moza, David Fish, Rachel J Peterson

**Affiliations:** 1 Internal Medicine-Pediatrics, Providence St. Vincent Medical Center, Portland, USA; 2 Internal Medicine-Pediatrics, University of Massachusetts, Worcester, USA; 3 Internal Medicine-Pediatrics, Indiana University School of Medicine, Indianapolis, USA

**Keywords:** med peds, hospital med, hospital based, med-peds, physician workforce

## Abstract

Objective

This article aims to describe the workplace characteristics of internal medicine and pediatrics (med-peds) hospitalists practicing hospital medicine (as internal medicine hospitalists, pediatric hospitalists, or both) in the United States.

Methods

The investigators conducted a cross-sectional survey of med-peds hospitalists via distribution through online platforms supported by the Society of Hospital Medicine (SHM), the American Academy of Pediatrics (AAP), and Twitter™. This sample was then reviewed and evaluated for similarities and differences in workplace characteristics.

Results

One hundred and sixteen respondents completed the survey and provided data on 63 unique institutions employing med-peds hospitalists. Of these institutions, 46% (n=29) employed six or more med-ped hospitalists within their hospital system. Furthermore, 44% (n = 28) of the institutions utilized the med-peds skillset to meet patient care needs in their hospitals. Forty hospitalists from 24 unique institutions saw both adults and children on the same day. Only 5.6% (n=6) of respondents were fellowship-trained. Interestingly, 34.9% of institutions (n=22) were required to provide adult-based care (age >21 years) within the pediatric hospital due to the COVID-19 pandemic. Of note, 35.5% (n=38) of participants from 24 unique institutions stated a high likelihood of hiring additional med-peds hospitalists in the next one to two years.

Conclusions

Med-peds hospitalists have a unique role within the hospitalist workforce given the variety of practice patterns and clinical needs they can fill within a hospital system. This survey provides the first sampling of workplace characteristics for actively practicing med-peds hospitalists in the United States.

## Introduction

There is limited literature on the varied employment structure of the combined internal medicine and pediatrics (med-peds) hospitalist workforce. As a product of double-board certification, med-peds physicians can enter a large variety of careers in academic, community, and global settings, ranging from subspecialties focused on conditions of childhood to hospital medicine or primary care. In recent studies, it is estimated that as many as 26.4% of med-peds graduates enter the field of hospital medicine [1]. In a 2015 American Association of Medical Colleges (AAMC) survey of med-peds physicians, 22.4% reported practicing as hospitalists, with a majority being early-career physicians practicing for less than 10 years. Over 80% of these clinicians saw both adults and children in their practice [2,3]. These sampled data demonstrate the ongoing growth of med-peds graduates who are seeking a defined hospitalist role involving both adult and pediatric care.

Med-peds hospitalists have also faced other factors impacting their career decisions and work structure [4]. The recent subspecialty certification recognition of pediatric hospital medicine in 2016 has impacted those practicing or entering the field of hospital medicine [5]. While over 97% of residents have considered a career in hospital medicine, subspecialty designation decreased their desire to pursue a career, including pediatric hospital medicine, for 90% of respondents [6]. In addition to the changes in graduate medical training, the COVID-19 pandemic has had a significant impact on current practice patterns and demands [7]. Med-peds physicians played a large role in the development of a network that aimed toward organizing clinical responses to the inpatient surge as well as addressing healthcare inequities [8].

The growing population of practicing hospitalists along with the factors mentioned above necessitates a further examination of the current field of med-peds hospital medicine. Prior publications have identified the demographics of the med-peds workforce. This publication aims to describe the current workplace trends of practicing med-peds hospitalists.

## Materials and methods

Survey design

We developed a 17-question survey for med-peds hospitalist physicians based on a review of the literature and an iterative consensus process with experts in the med-peds workforce. This survey sought to assess the workplace characteristics of med-peds hospitalists across the United States. Respondents were asked about the practice patterns and operational structure of hospitalist program(s) within their institutions that were staffed by med-peds physicians. We also included questions surrounding the impact of the COVID-19 pandemic on changes in clinical work for med-peds hospitalists.

Setting, administration, analysis

The survey was dispersed from March 2021 to June 2021 via multiple electronic mailing lists (listservs), including the online platform for SHM known as HMx™, Twitter™, and the American Academy of Pediatrics (AAP) Society of Hospital Medicine (SHM) med-peds mailing list. The lists were reviewed by the authors to prevent duplicate responses. The cycle of dispersal through organizational listservs was repeated four times.

Responses were excluded if surveys were left incomplete. After this initial filtration, the results were analyzed using the statistical software Microsoft Excel Version 16.16.27 (Microsoft® Corp., Redmond, WA). Responses received from each unique institution were manually reviewed as related to operational characteristics. The Providence Institutional Board Review exempted this survey protocol from additional review.

## Results

We received 116 total responses, of which 107 met inclusion criteria.

Hiring structure

This survey identified at least 63 unique institutions employing med-peds hospitalists. Of these, 46.0% (n=29) support hiring through the Department of Medicine and 17.4% (n=11) support hiring through the Department of Pediatrics, while 20.6% (n=13) support hiring through the academic institution instead of a singular Department of Medicine or Pediatrics. Hospital-based employment was reported by 23.8% (n=15) of institutions. The total percentages add up to over 100% due to 7 of 63 institutions providing hiring support through more than one venue (Table 1).

**Table 1:** Location of hiring and support of benefits structure

Distribution	N	Percentage of total (N=63)
Department of Medicine	29	46.0%
Department of Pediatrics	11	17.4%
Hospital-based system	15	23.8%
Other	13	20.6%

Clinical practice and expertise

From the total number of respondents, 37% (n=40) from 24 unique institutions clinically provide care to both adults and children on the same clinical day, and 44.8% (n=48) alternate between the two roles of pediatric and adult hospital care. There was a smaller portion of med-peds trained hospitalists, 6.5% (n=7), practicing hospital medicine in either medicine or pediatrics, but not both. The remaining 11.2% (n=12) reported practicing in a combined structure of inpatient and outpatient care. Only 5.6% (n=6) were fellowship-trained, of which half of those, 2.8% (n=3), were in pediatric hospital medicine.

Out of these 63 unique institutions, 25.3% (n=16) housed a consulting service uniquely staffed by med-peds hospitalists. Out of the 107 respondents, 34.5% (n=37) stated that the med-peds hospitalists provided unique expertise or staffed the adults on the pediatric units (Table 2). When reviewed on an institutional basis, 44% (n=28) of institutions reported that the med-peds skillset was being used to "meet a specific patient care need."

**Table 2:** Presence of services uniquely staffed by med-peds hospitalists

Service identified	N	Percentage of total institutions
Consult service	16	25.3%
Unique staffing of adults hospitalized	28	44%
None reported	19	30.2%

Impact and growth

Of the surveyed institutions, 11.4% (n=7) reported having a formal med-peds hospitalist division, and 46% (n=29) of institutions employ more than six med-peds hospitalists within their hospital system. Of the respondents, 35.5% (n=38; representing 24 unique institutions) stated a high likelihood of hiring additional med-peds hospitalists in the next one to two years.

In the context of the COVID-19 pandemic, this survey sought to evaluate clinical practice changes regarding the hospitalization of adults on pediatric floors. Thirty-one institutions had pediatric hospitals admitting adults >21. During the pandemic, 22 pediatric institutions (34.9% of total unique institutions) were required to adapt to adult-based care within an operational year.

## Discussion

This survey describes the institutional characteristics and practice patterns of actively practicing med-peds hospitalists. An estimated 100 new med-peds hospitalists join the workforce each year [9], with an estimated total number of practicing med-peds hospitalists in 2015 at around 350-400 total [2]. Based on the reported numbers of hospitalists per group from this study (approximately 250 on a low estimate), this survey encompasses a large proportion of the estimated total of those practicing med-peds hospitalists.

Our results are in line with prior surveys outlining that a majority of med-peds physicians practice both adult and pediatric hospital medicine [2,3,10]. The growing number of practicing med-peds hospitalists demands a better understanding and distinction of the unique needs of these doctors in order to better identify career opportunities and areas for improvement. Prior literature reviewed the combined med-peds hospital medicine workforce via the lens of graduate medical education [2,3], and current trainee perspectives [6]; this survey adds the perspective of the active med-peds hospitalist workforce.

Adult-aged patients with childhood-onset conditions make up a growing presence at pediatric hospitals [11-15]. Med-peds hospitalists provide clinical expertise in addressing the adult and pediatric needs of this population. Future studies to evaluate the best methods for ongoing medical education and professional networking are needed to ensure the success of patient care and population health.

There is increasing demand for med-peds hospitalists reported in this study, based on reported plans for expansion within many hospitalist groups. The adaptability of this workforce reflects needs from census shifts and practice changes forced by the COVID-19 pandemic [7,8]. In setting of increased demand for med-peds trained hospitalists, the requirement for PHM fellowship training may deter trainees from pursuing a career in hospital medicine - just at the time that this adaptability is needed most. As the field continues to evolve, future studies will need to further evaluate the impact these changes have on the med-peds workforce regarding the number of graduates who enter hospital medicine and whether these providers decide to pursue PHM fellowship. The future requirements for pediatric hospitalist fellowship training may impede the presence of med-peds hospitalists who have a unique skill set not provided by either of the categorical specialties [6].

It is interesting to note that, in reviewing the structure of med-peds hospitalist jobs, many respondents stated that their benefits and primary appointments are housed within the Department of Medicine. While this survey did not investigate the reasons behind program structure, differences in fiscal support for hospitalist positions through internal medicine departments may be due to increased profitability of adult (versus pediatric) care as one possible contributor. Financial flexibility might allow med-peds hospitalists to navigate alternative compensation structures and clinical obligations when compared with their categorical counterparts [4].

As the first study to classify the workplace characteristics of currently practicing med-peds hospitalists this study has several limitations. Given the voluntary response, there may be a lack of representation of the total number of combined med-peds hospitalists practicing in the United States. While we attempted to query an even number of adult and pediatric-based societies, due to the lack of a national med-peds organization for practicing physicians, a true comparator of response rates cannot be calculated. Finally, non-response bias may indicate a skew of data over-estimating the prevalence of med-peds hospitalists clinically practicing both adult and pediatric hospital medicine and underestimating the number of med-peds trained hospitalists who care for only children or adults.

This paper provides the first overview of workplace characteristics outlining the med-peds hospitalist workforce. Future studies will be critical to evaluating the longitudinal changes and sustainability of this workforce.

## Conclusions

Med-peds hospitalists have a unique role within the hospitalist workforce. The full extent of dual-trained hospitalist physicians in US practice is difficult to ascertain as these providers may belong to multiple medical societies pertaining to pediatric, adult, and hospital-focused practice. However, this survey better outlines the current practice of these dual-trained providers. They fill a variety of practice patterns and clinical needs within a hospital system and meet the needs of growing numbers of vulnerable patients in pediatric and adult health systems. The workplace characteristics described in this study provide a foundation to build additional support and future studies looking at this unique population of physicians.
